# Connective tissue metabolism in chikungunya patients

**DOI:** 10.1186/1743-422X-5-31

**Published:** 2008-02-27

**Authors:** Sudarsanareddy Lokireddy, Sarojamma Vemula, Ramakrishna Vadde

**Affiliations:** 1 Department of Biotechnology, Sri Krishnadevaraya University, Anantapur, India; 2 Department of Community Medicine, Government Medical College, Anantapur, India

## Abstract

**Background:**

Chikungunya (CHIK) fever is a viral disease transmitted to humans by the bite of Chikungunya virus (CHIK virus) infected *Aedes *mosquitoes. CHIK virus is a member of the *Alphavirus *genus of the family *Togaviridae*. Previous reports have indicated that infection with CHIK virus produces an acute arthritis in human hosts by large area of necrosis and collagenosis or fibrosis.

**Results:**

We carried out the present study to determine the effect of chikungunya on the collagen and connective tissue metabolism in 75 chikungunya-affected people. First, we screened for mucopolysaccharides in urine by Cetyl Trimethyl Ammonium Bromide (CTAB) test. Appearance of heavy precipitate indicates the presence of higher levels of mucopolysaccharides and later quantified by DMB dye method. The urinary mucopolysaccharide in CHIK patients was 342 ± 45 mg/l compared to healthy controls (45 ± 5.6 mg/l). The collagen building blocks, proline and hydroxyproline were also measured in CHIK patients and observed higher excretion compared to healthy controls. Urinary excretions hydroxyproline was greater than the proline levels.

**Conclusion:**

These results indicate that CHIK virus infection affects and damage the cartilage and connective metabolism and releases the degraded products from the tissue and responsible for increasing the levels of proline, hydroxyproline and mucopolysaccharides in CHIK affected patients.

## Background

Chikungunya (CHIK) fever is a viral disease transmitted to humans by the bite of Chikungunya virus (CHIK virus) infected *Aedes *mosquitoes. CHIK virus is a member of the *Alphavirus *genus of the family *Togaviridae*. CHIK virus was first isolated from the serum of a febrile patient during a dengue epidemic that occurred in the Newala District, Tanzania in 1953 [1]. The *Alphaviruses *are enveloped particles and their genome consists of a single-stranded, positive-sense RNA molecule of approximately 12000 nucleotides. CHIK virus is an important human pathogen that causes a disease syndrome characterized by fever, headache, rash, nausea, vomiting, myalgia and arthralgia [2-8]. Its association with a fatal haemorrhagic condition was reported in India [9]. CHIK virus is geographically distributed from Africa through Southeast Asia, and its transmission to humans is mainly through *Aedes *species mosquitoes [1]. Since 1953, CHIK viurs has caused numerous well-documented outbreaks and epidemics in both Africa and Southeast Asia, involving hundreds of thousands of people [10-12]. Recent reports have described a massive outbreak of CHIK disease occurring on islands in the Indian Ocean, off the east coast of Africa [5,8,13]. Reemergence of CHIK has also been reported from Indonesia [14] and very recent massive outbreak in India. No treatment or vaccine is available, and relatively little research has been conducted into its pathogenesis, compared with that of other *Arboviruses*, such as dengue.

Previous reports have indicated that infection with CHIK virus produces an acute arthritis in human hosts [7,15,16] by large area of necrosis and collagenosis or fibrosis [17]. Arthritis is the inflammation of a joint and its surrounding tissues, including the cartilage, ligaments, and tendons. Connective tissue is mainly composed of mucopolysaccharides, protein substances – collagen with major amino acids proline, hydroxyproline etc, calcium, sulfur, and collagen. Mucopolysaccharide, proline and hydroxyproline measurements in urine are used in the diagnosis and treatment of various inheritable disorders that affect bone and connective tissues. Their presence in urine is originated from ground substance of collagen, and bone [18-20]. Though many reports state that CHIK virus produces arthritis in humans. The biochemical mechanisms and the changes of mucopolysaccharides, proline and hydroxyproline in this acute joint pain have not been reported in past literature. During last year monsoon outbreak of CHIK fever in Southern parts of India has led us to carryout the experiments on the effect of CHIK virus on the collagen and connective tissue. In this study we collected the blood and urine samples from chronic CHIK fever patients and studied biochemical parameters related to connective tissue, which is responsible for acute joint pains and arthritis.

## Results

CHIK patients have reported incapacitating joint pain, or arthritis, which may last for weeks or months. The general biochemical parameters of CHIK patients and healthy controls were depicted in Table 1. Serum albumin and creatinine were showing significant variation with controls. We carried out the present study to determine the effect of CHIK on collagen and connective tissue metabolism by measuring the levels of mucopolysaccharide, hydroxyproline and proline in urine samples of CHIK patients. Urine from 75 different patients suffering with CHIK was evaluated for the presence of mucopolysaccharides by screening cetyl trimethyl ammonium bromide (CTAB) test in urine. Appearance of heavy precipitate indicates the presence of mucopolysaccharides in the sample. The amount of precipitate depends on the concentration of mucopolysaccharides. Depending upon their concentration, samples were classified as negative, mild, moderate, and severe. In this study, we observed moderate to severe cases. The healthy controls showed no precipitate with CTAB. We also measured quantitatively urinary mucopolysaccharides levels in the first morning urine specimens by the simple and rapid DMB method and used in understanding the pathophysiology of arthritis in CHIK diseases. Urinary mucopolysaccharides (GAG) excretion in patients increased significantly compared to healthy controls. We also observed the significant variation in the levels of urinary excretions of hydroxyproline and proline with healthy controls (Table 2). The patients suffering with CHIK excreted higher levels of hydroxylproline than proline. We also carried out the follow up study for 10 patients with their consent up to one week who has chronic arthritic pains. The urinary excreted mucopolysaccharides are continuously high in the first two days and the levels were slowly decreasing on later days, but at the end of one week (up to 10 days) we found still the patients are excreting higher values compare to control (Table 3). The changes in the levels of urinary hydroxyproline and proline were also measured and observed the significant difference with healthy controls.

**Table 1:** Biochemical characteristics of normal and Chikungunya patients

**S. No**	**Clinical data**	**Normal**	**Chikungunya**
1.	Number	20 F – 6, M – 14	75 F – 30, M – 45
2.	Age (years)	20 – 50	15 – 60
3.	Blood glucose	80 ± 12 mg/dl	90 ± 16 mg/dl
4.	Serum total protein	7.15 ± 0.96 g/dl	6.96 ± 0.6 g/dl
5.	Serum albumin	4.04 ± 0.45 g/dl	3.60 ± 0.24 g/dl^$^
6.	A:G ratio	1 – 2	0.96 ± 0.4
7.	Blood urea	36 ± 8 mg/dl	41 ± 10 mg/dl
8.	Serum creatinine	0.96 ± 0.23 mg/dl	1.22 ± 0.12 mg/dl^$^

F-Female, M-Male, mg- milli gram, dl- deci litre.

Results are expressed as mean ± SD. ^$ ^p < 0.05 against the control values

**Table 2:** Effect of chikungunya (on average day 3 – 5) on the levels of proline, hydroxyproline and acid mucopolysaccharides

**Sl No**.	**Parameter**	**Control**	**Chikungunya**
1.	Proline	1.4 ± 0.18 mg/l	22 ± 3.2 mg/l^$^
2.	Hydroxyproline	25 ± 1.8 mg/l	210 ± 34 mg/l^$^
3.	Mucopolysaccharides	45 ± 5.8 mg/l	342 ± 45 mg/l^$^

Results are expressed as mean ± SD. ^$ ^p < 0.05 against the control values

**Table 3:** Changes of urinary proline, hydroxyproline and mucopolysaccharides during week long Chikungunya fever.

**Day**	**Proline mg/l**	**Hydroxyproline mg/l**	**Mucopolysaccharides mg/l**
Day 1	32 ± 2.1	263 ± 22	380 ± 45
Day 2	30 ± 1.6	251 ± 43	372 ± 30
Day 3	25 ± 2.0	228 ± 38	354 ± 26
Day 4	20 ± 1.8	210 ± 24	325 ± 28
Day 5	16 ± 2.0	186 ± 15	300 ± 30
Day 6	15 ± 1.5	160 ± 13	260 ± 22
Day 7	10 ± 0.8	138 ± 11	221 ± 18
Day 8	10 ± 0.5	130 ± 10	210 ± 16
Day 9	9 ± 0.8	120 ± 14	185 ± 10
Day 10	7 ± 0.54	100 ± 10	160 ± 12

## Discussion

Proteoglycan, a mucopolysaccharide building block of cartilage within the joint space, is used by chondrocytes (cartilage-building cells) to create more cartilage. Following pathogenic infection, joints typically exhibit a natural inflammatory response that mainly affects the synovium (synovitis). This process is necessary for the innate repair of damaged tissues, allowing the joint to recoup normal function. Inflammatory mediators released into the joint from such sources as nerves, immunocytes, synoviocytes, and vascular endothelium help to orchestrate these healing responses. These same inflammatory mediators also act on joint sensory nerves, leading to either excitation or sensitization [21]. When people suffer from arthritis, they experience a selective destruction of collagen within the cartilage of their joints. Collagen is the most abundant structural protein in cartilage. It helps provide elasticity of joints and works as the "glue" that holds together the various components of cartilage. It also contains the majority of mucopolysaccharides that give cartilage its healing powers. Mucopolysaccharides are large-molecular mass linear carbohydrate polymers composed of glucuronic or iduronic acid and N-acetylglycosamine or N-acetylgalactosamine units. Chondroitin sulfate and heparan sulfate are derived from proteoglycans in which these glycosaminoglycans (GAG) are covalently linked at the reducing end to the hydroxyl group of serine residues [22]. Proteoglycans containing GAG bound to hyaluronic acid form large aggregates that are enmeshed in a collagen network as the principal structural constituents of cartilage. Loss of proteoglycans is an early feature of matrix resorption. The erosion of the cartilage lining the joints is a hallmark of osteoarthritis. A corresponding increase in GAGs has been reported in synovial fluid, serum, and urine of patients with rheumatic arthritic diseases [23,24]. Consistent with this, we observed in our studies higher excretion of mucopolysaccharides through urine (Table 2).

Measurement of urinary hydroxyproline excretion is used to estimate collagen catabolism. Degradation of bone collagen releases free 4-hydroxyproline and peptides containing 4-hydroxyproline into the plasma. Urinary hydroxyproline measurement is particularly useful in monitoring treatment and progress in metabolic bone diseases such as osteomalacia and osteoporosis and Paget's disease. Hydroxyprolme appears in urine after degradation of collagen (collagenosis), which may originate from various tissues [25]. Measuring urinary hydroxyproline has predominantly been used to indicate bone collagen turnover in normal and pathological conditions. Collagen, which constitutes >90% of the fat-free organic matrix of bone, is important in its structural integrity and is the central element of connective tissue structure in which the mineral phase crystallizes during bone formation [20,26]. Urinary hydroxyproline reflects daily collagen turnover and in humans has shown great potential as an index of nutritional status and growth rate [27,28]. The CHIK patients showed higher excreted levels of hydroxyproline than proline indicates increased collagen turnover (Table 2). On follow-up study in the selected CHIK patients, increased levels of mucopolysacchariedes, proline and hydroxyproline indicates highermetabolic turnover in early days and recedes progressively in later days (Table 3).

## Conclusion

In CHIK patients the collagen and connective tissue metabolism was greatly affected and increased metabolism leads to increased excretion of proline, hydroxyproline and mucopolysaccharides in urine compared to healthy controls. It indicates that the CHIK virus infection, connective tissue typically exhibits inflammatory response and leads to the damage of cartilage and connective tissue; increases metabolism and releases degraded products in to blood and ultimately excreted through urine.

## Materials and methods

### Subjects

During last year July – October 2006, CHIK out break in Andhra Pradesh, India, a total of 75 (45 males + 30 females) patients were selected based on disease symptoms for this study. We also taken the 20 age-matched healthy persons were chosen from the community and used as control subjects. Blood and urine samples were collected from each subject with their prior written consent for further studies. From all subjects, 5 ml of blood was collected in heparinized tubes and centrifuged at 1500 ×g for 15 min at 4 C. Plasma and pelleted RBC were separated stored in eppendorf tubes and kept at -80 C until analysis. Commercially available kits measured Blood analysis and other general parameters.

### Urine analysis

The urine samples were collected from the patients and used for estimation of mucopolysaccharides, proline and hydroxyproline. Total urinary hydroxyproline determinations were performed employing the method described by Cleary and Saunders (1974) [29]. Briefly, urine was passed through a column of ion retardation resin to remove acid, salts, and pigments. From the column eluate, urine hydroxyproline determination was performed by oxidized to pyrrole 2-carboxylic acid and then to pyrole. The color produced with dimethyl amino-benzaldehyde is masured at 560 nm [30]. The proline levels in urine sample were measured by the method of Bates et al (1973) [31]. Two ml urine sample mixed with 2 ml acid ninhydrin reagent and kept in boiling water bath for 1 h. The tubes transferred to an ice bath to terminate the reaction. 5 ml toluene was added and mixed and read at 520 nm against the toluene blank. Screening test for mucopolysaccharides in urine was tested by Cetyl Trimethyl Ammonium Bromide (CTAB) test. Five ml of fresh urine was added to 1 ml of CTAB (cetavilon) solution (50 g/l in citrate buffer (1 M) of pH 6.0). A heavy precipitate indicated the presence of mucopolysaccharides (Varley et al 1990). The DMB assay was performed essentially for measurement of mucopolysaccharides according to the method of Whitley et al. (1989) [32]. Briefly, the DMB (1,9-dimethylmethylene blue) dye solution was prepared by dissolving 16 mg of DMB, 3.04 g of glycine, 2.37 g of sodium chloride, and 0.5 mL of 0.1 mol/l hydrochloric acid in 1 l of distilled water. The pH of the solution was adjusted to 3. For each assay, the patient's urine specimen was mixed with 1 ml of the dye solution at room temperature, and measured absorbance at 525 nm without delay after mixing the sample and dye solution. The assay was calibrated verses a standard curve of chondroitin sulfate (5–100 mg/l). The results were expressed as mg/l. All colorimetric estimations of above biochemical parameters in samples were measured by using BIOTEK ELISA Reader.

### Statistical analysis

Statistical analysis was performed by GraphPad InStat software. Subjects with CHIK were compared with healthy controls. Means and standard error of means were calculated and differences between means were student's t-test.

## Authors' contributions

SL designed and conducted the experiments with patient's blood, urine samples. SL also performed the final statistical analysis of the data and contributed to writing the paper. SV (she is specialist in infectious diseases) guide to us for selecting the chikungunya patients for research during time of blood and urine collection. RV supervised the overall project, designed experiments, analyzed the data and wrote the paper. All authors read and approved the final manuscript.
